# Measuring inequalities in the public health workforce at county-level Centers for Disease Control and Prevention in China

**DOI:** 10.1186/s12939-019-1073-4

**Published:** 2019-11-21

**Authors:** Weiqin Cai, Chengyue Li, Mei Sun, Mo Hao

**Affiliations:** 1 Research Institute of Health Development Strategies & Collaborative Innovation Center of Social Risks Governance in Health, Fudan University, 177 box, 130 Dong’an Road, Shanghai, 200032 China; 2 Department of Health Economics, School of Public Health and Management, Weifang Medical University, Weifang, Shandong China

**Keywords:** Public health workforce, Inequalities decomposition, CDC, County-level

## Abstract

**Background:**

The public health workforce (PHW) is a key component of a country’s public health system. Since the outbreak of SARS (severe acute respiratory syndrome) in 2003, the scale of PHW in China has been continuously expanding, but policymakers and researchers still focus on the distribution of public health personnel, especially the regional inequality in such distribution. We aimed to identify the root cause of PHW inequality by decomposing different geographical units in China.

**Methods:**

This study was based on data from a nationwide survey, which included 2712 county-level data. The distribution of the PHW in geographical units was evaluated by the Gini coefficient and Theil T index, and inequalities at regional, provincial, and municipal levels were decomposed to identify the root causes of inequalities in the PHW. Additionally, the contextual factors affecting the distribution of the PHW were determined through regression analysis.

**Results:**

The overall inequality results show that health professional and field epidemiological investigators faced worse inequality than the staff. In particular, field epidemiological investigators had a Gini coefficient close to 0.4. Step decomposition showed that within-region inequalities accounted for 98.5% or more of overall inter-county inequality in the distribution of all PHW categories; provincial decomposition showed that at least 74% of inequality is still distributed within provinces; the overall contribution of within-municipal inequality and between-municipal inequality was basically the same. Further, the contextual factor that influenced between-municipality and within-municipality inequality for all three categories of PHWs was the agency building area per employee. Per capita GDP had a similar effect, except for between-municipality inequality of professionals and within-municipality inequality of field epidemiological investigators.

**Conclusions:**

The successive decomposition showed that inequality is mainly concentrated in counties at the within-province and within-municipal levels. This study clearly suggests that the government, especially the municipal government at the provincial level, should increase financial investment in Centers for Disease Control and Prevention (CDCs) with worse resource allocation in their jurisdiction through various ways of compensation and incentives, enhance their infrastructure, and improve the salary of personnel in these institutions, to attract more public health professionals to these institutions.

## Introduction

The public health workforce (PHW) is the key component of a nation’s public health system [1, 2]. Thoroughly characterizing and continuously monitoring the quantity and capabilities of the PHW ensures the ability to provide public health services [3]. Since 2000, countries around the world have been strengthening their public health systems to address new infectious diseases, terrorism, and other public health threats [4]. Accordingly, the investment and scale of public health manpower have greatly increased [5, 6]. For example, the United States published two reports on PHW development, reporting that funding for PHW preparedness and training has increased since 2002 [7].

While developing their PHWs, many countries have begun to value the research and analysis of existing PHWs to ensure an adequate PHW and identify the high-level professional and technical personnel with core competitiveness [8]. While the scale of the PHW has grown, concerns remain regarding the composition, distribution, skills, and performance of the PHW. Simultaneously, different geographic areas of a country experience general inequality in the distribution of public health services [9, 10]. The pursuit of health equity means striving for the highest possible standard of health for all and paying special attention to the needs of those in poor health based on their geographical distribution [11].

After 40 years of reform, China’s economic and comprehensive national strength have achieved breakthroughs, and resource allocation in all aspects has been strengthened overall; however, many structural problems have accumulated following years of sustained “rapid development.” A recent document shows that China has focused on the “unbalanced and inadequate” development and addressed it as a major social conflict [12], which applies to the public health field.

Since the outbreak of severe acute respiratory syndrome (SARS) in 2003, China has established an effective public health system and invested significant human, financial, and material resources [13]. Regarding PHW allocation, our previous research found that the number of PHWs in Centers for Disease Control and Prevention (CDCs) increased after the outbreak of SARS [13]. In China, CDCs are an important component of the public health system and are the core force for preventing and controlling health threats [14, 15]. The main functions of CDCs include disease prevention and control, disease monitoring and investigation, health education and promotion, research and guidance, and technical management and service. In mainland China in 2012, there was a four-tiered Disease Control and Prevention System, which contained one national, 31 provincial (including five autonomous regions and four municipalities), 333 municipal (including 285 prefecture-level cities, 30 autonomous prefectures, 15 districts, and 3 leagues), and more than 2852 county-level CDCs [16]. In a country with a large population and a wide geographic area, public health personnel is distributed over a sizable system. Therefore, one issue that is of relevance is its geographical distribution, especially in the most basic geographical units. Regarding policy-making related to health equity, it is crucial to identify the geographical distribution characteristics of PHWs in China and to determine the sources and contextual factors of PHW inequality. This can also simultaneously contribute to enriching the literature on PHW distribution factors in similar developing countries.

In 2010, the World Health Organization (WHO) reported that current measurement of inequality does not make full use of human resources data [17]. Furthermore, it gives an inequality measure of health workers using the smallest comparable geographical units of data in a nation. Subsequent studies have applied this method and made good progress [11, 18], but none have been completely followed because of the unavailability of data nationwide. At the same time, current research on China mainly concerns equity analysis of the health workforce and includes little relevant research of the PHW [19]. This study aimed to assess the distribution of PHWs within geographic units based on the availability of country-wide survey data as well as to identify the sources of PHW inequality through the decomposition of inequality, so as to provide a reference for the development of human resource strategies.

An understandable fact is that most public health workers prefer to settle in developed areas where they can provide for themselves and their spouses and children and have more opportunities for professional development, education, and other conveniences. However, in rural and remote areas where the most serious public health problems have been identified, especially in developing countries, PHWs are scarce. As a consequence, there is a growing number of studies that have also focused on the influencing factors of health resource distribution [20], especially the determinants of variations in geographical distribution [21]. The factors that motivate, promote, and retain public health workers move to areas offering attractions such as better salaries and training opportunities mainly constitute financial, career development, continuing education, institutional infrastructure, resource availability, organization management, personal recognition or appreciation, and social elements including educational opportunities for children and employment opportunities for spouses [22]. Therefore, based on an inequality analysis and decomposition of inequality, the present study attempted to determine the contextual factors of PHW inequality to analyze the causes of inequality in PHW distribution.

## Methods

### Data

We used data from a national cross-sectional survey of the CDC system for 2012. We conducted this survey in partnership with the Ministry of Health and 31 provincial departments of health in July–October 2013. As we previously reported [13, 23], to ensure data quality, the Ministry of Health trained provincial quality supervisors in questionnaire administration, and then the Provincial quality supervisors delivered the surveys to investigators from CDCs at the municipal and county levels. The research team was responsible for checking the data quality and checking abnormal values by phone or email.

In China, “county-level” refers to counties, autonomous counties, county-level cities, qi, autonomous qi, and districts. Henceforth, county refers to these county-level units. After removing missing values, we included 2712 counties in the analysis. Household registration data for 2012 was the source of the county-level population data (Ministry of Public Security, China, 2013).

We examined three categories of the PHW: (1) staff; (2) health professionals; and (3) field epidemiological investigators. These categories are not discrete, as health professionals are a subset of staff, and field epidemiological investigators are a subset of health professionals. Staff refer to all the people working for the CDC, both permanent and temporary workers. Health professionals refer to people with specific health expertise and corresponding operational skills, including practicing physicians (which includes public health physicians), assistant practicing physicians, registered nurses, pharmacists, and inspection technicians. We did not include health technical personnel engaged in management work (e.g., directors, deputy directors, and secretaries of the party committee of the CDCs). Field epidemiological investigators refer to those who have obtained professional qualifications in epidemiology or technical qualifications in medically related fields, and have been trained in field epidemiological investigations.

### Analysis of inequality

Three indicators of inequality can be used to measure inequalities in the distribution of public health workers to explain the causes of a country’s inequality. The first indicator is the Gini coefficient, the most widely used measure of inequality, although it cannot be disaggregated and therefore cannot explain the sources of inequality. The second indicator is the Theil L index, which is considered to be the most attractive decomposition indicator, as it can be decomposed into two parts, between-groups and within-groups. The third indicator is the Theil T index, which is an entropy index that can be decomposed and that can allow the inclusion of subset geographical units with zero health workers.

This study selected the Gini coefficient and Theil T index to measure inequality in the three categories of PHWs. The WHO has identified both approaches to measure inequality in the distribution of health workers and to explain the sources of inequality, while also giving a detailed derivation process and formula [24]. We chose Theil T instead of Theil L because there were no field epidemiologists in a few county-level CDCs.

Suppose the sample containing *n* individuals is divided into *K* groups, each group is *g*_*k*(*k* = 1, 2, 3…*K*)_, the number of individuals in *g*_*k*_ of group *k* is *n*_*k*_, then $\sum \limits_{k=1}^k{n}_k=n$, *y*_*i*_ and *y*_*k*_ represent the human share of an individual *i* and the total human share of a group *k*, respectively. *T*_*b*_ and *T*_*w*_ represent the between-group differences and within-group differences, respectively, so the decomposition of Thiel T index is as follows:

$$ T={T}_b+{T}_w=\sum \limits_{k=1}^K{y}_k\mathit{\log}\frac{y_k}{n_k/n}+\sum \limits_{k=1}^K{y}_k\left(\sum \limits_{i\in {g}_k}\frac{y_i}{y_k}\mathit{\log}\frac{y_i/{y}_k}{1/{n}_k}\right) $$

### Statistical analysis

A regression analysis was conducted to understand the contextual factors that might affect the within-group and between-group inequality of PHWs. This regression analysis was only performed for the municipal level. All 333 municipal-level CDCs were included in the fitted linear regression model. In the model, the inequality values between and within the three PHWs were taken as dependent variables. Since the decomposition value of the Theil index can have both negative and positive values, we transformed the between-municipality and within-municipality inequality values to only positive values from 0 to 1. The higher the value of the transformed variable, the higher the share of PHWs. We used the following formula used in similar studies to make this transition:

Theil Transformed = (actual value−minimum value)/(maximum value−minimum value) [10].

The contextual variables used in the regression analysis were derived from the general baseline data questionnaire, as well as questionnaires on the financial receipts and expenditures, and on infrastructure for the county-level CDCs. These variables were selected based on the existing literature on factors determining the geographical distribution of human resources [20–22], including: i) per capita GDP, which represents the socio-economic factors that may contribute to the disparity in the PHWs’ distribution; ii) agency government financial allocation per employee, which is a financial factor that contributes to the wages and benefits for PHWs; iii) agency building area per employee, which has implications on the institutional infrastructure, and also can be described as “work environment”; iv) numbers of training days per employee per year, which represents the opportunity for continuing education and future development; and v) numbers of kindergartens and primary schools per 10,000 population, which represents the impact of social conditions on families (especially children).

County-level data are used to calculate various municipal-level variables. Therefore, our unit of analysis is the municipality. Using county-level data, we calculated five variables at the municipal level. In the regression model, we controlled for regional variables based on the level of economic development to explain the significant regional differences in PHW availability.

The inequality analyses were completed using Microsoft Excel 2013 (Microsoft Corporation, Redmond, WA, USA), while the analysis of descriptive data and regression analysis were performed using SPSS 22.0 (SPSS Inc., Chicago, USA). ArcGIS software (Redlands, California, USA) was used to map the inequality of the geographical distribution of PHWs.

## Results

### Overall inequality of PHWs

Overall inter-county inequalities in the distribution of all three categories of PHWs (Table 1) were not the same, with a Gini of 0.2948 for staff (0.0657 for Theil T), 0.3038 for health professionals (0.0703 for Theil T), 0.3788 for field epidemiological investigators (0.1089 for Theil T). There was higher overall inequality in absolute terms in the distribution of field epidemiological investigators than in that of staff or health professionals for both Theil T and Gini.

**Table 1:** Density and inter-county inequality (*n* = 2712) by region^a^ for three categories of PHWs in China, 2012^b^

Workforce Category		East	Central	West	All Regions
		(*n* = 865)	(*n* = 860)	(*n* = 987)	(n = 2712) ^c^
Staff ^d^	Number	45,985	47,224	40,616	133,825
	Mean density	0.94	1.05	1.06	1.01
	Min county density	0.01	0.11	0.09	0.01
	Max county density	12.89	17.87	17.03	17.87
	Theil T	0.0553	0.0647	0.0756	0.0657
	Gini	0.2726	0.2964	0.3125	0.2948
Health professionals ^d^	Number	36,639	33,922	33,296	103,857
	Mean density	0.75	0.76	0.87	0.79
	Min county density	0.01	0.06	0.63	0.01
	Max county density	12.89	13.66	13.08	13.66
	Theil T	0.0578	0.0686	0.0821	0.0703
	Gini	0.2772	0.3056	0.3263	0.3038
Field epidemiological investigators ^d^	Number	18,643	17,938	16,320	52,901
	Mean density	0.38	0.40	0.43	0.40
	Min county density	0.00	0.00	0.00	0.00
	Max county density	1.78	7.36	8.72	8.72
	Theil T	0.0848	0.1025	0.1414	0.1089
	Gini	0.3385	0.3725	0.4279	0.3788

Abbreviation: *PHW*, Public health workforce

^a^ According to the level of economic development, China is divided into eastern, central, and western regions. The eastern region is an economically developed region, including Beijing, Tianjin, Hebei, Liaoning, Shanghai, Jiangsu, Zhejiang, Fujian, Shandong, Guangdong, and Hainan, with a total of 11 provinces and independent municipalities; the central region is an economically developed region, including Shanxi, Jilin, Heilongjiang, Anhui, Jiangxi, Henan, Hubei, and Hunan, with a total of 8 provinces. The western region is an economically underdeveloped region, including Inner Mongolia, Chongqing, Guangxi, Sichuan, Guizhou, Yunnan, Tibet, Shaanxi, Gansu, Qinghai, Ningxia, and Xinjiang, with a total of 12 provinces, autonomous regions, and independent municipalities under the Central Government

^b^ Data source: National cross-sectional survey of the CDC system for 2012, Division of Disease Control and Prevention, Ministry of Health

^c^ There was 2852 county-level CDCs in mainland China in 2012, we included 2712 counties for analysis due to some missing data

^d^ These categories are not discrete, as health professionals are a subset of staff, and field epidemiological investigators are a subset of health professionals

### Decomposition of PHW inequalities

#### Regional-level contribution to overall inequality

Table 2 provides a decomposition of overall inter-county inequality into within- and between-region inequality. Within-region inequality accounted for at least 98.5% of overall inter-county inequality in the distribution of all workforce categories. For field epidemiological investigators, within-region inequality was highest at 99.5% (0.1084 of 0.1089) of overall inter-county inequality. Hence, in the distribution of PHWs, within-region inequalities caused almost all inter-county inequality.

**Table 2:** Decomposition of inter-county inequality (*n* = 2712) by region for three categories of PHWs in China, 2012^a^

Workforce category	Inequality measure	Overall inter-county inequality	Within- region inequality	Between- region inequality	Within- region inequality (% of overall)^b^	Between- region inequality (% of overall)^c^
Staff	Theil T	0.0655	0.0649	0.0008	98.9%	1.1%
	Gini	0.2948				
Health professionals	Theil T	0.0702	0.0692	0.0010	98.5%	1.5%
	Gini	0.3038				
Field epidemiological investigators	Theil T	0.1088	0.1084	0.0005	99.5%	0.5%
	Gini	0.3788				

Abbreviation: PHW, public health workforce

^a^ Data source: National cross-sectional survey of the CDC system for 2012, Division of Disease Control and Prevention, Ministry of Health. County number is 2712 because missing data

^b^ Within-region inequality (% of overall) is the ratio of “within-region inequality” to “overall inter-county inequality”, and between-region inequality (% of overall) is the ratio of “between-region inequality” to “overall inter-county inequality”

Table 1 summarizes the PHW distribution within regions and displays the number of counties and health workers, and the mean, minimum, and maximum county density per 10,000 population, and the three inequality measures. The overall density of CDC staff in China was 1.01 per 10,000 population, and the overall density of health professionals and field epidemiological investigators was 0.79 and 0.40 per 1000 population, respectively. The density of the three categories of PHW did not vary greatly between regions, and was similar to the national level. As the latter are a subset of staff and health professionals, the densities per 10,000 population were lower for field epidemiological investigators than for the other categories. For China as a whole, there were 0.40 field epidemiological investigators per 10,000 population, ranging from 0.38 in the West region to 0.43 in the East region.

The minimum and maximum county density of the three categories of PHWs are provided in Table 1. For staff, the East region had the lowest minimum county density, with 0.01 per 10,000 population, while its maximum was 12.89. The absolute minimum-maximum range was widest in the West region: 0.09 to 17.03 per 10,000 population. For health professionals, the lowest minimum county density was also in the East region, with 0.01 per 10,000 population, while its maximum was 12.89. However, the widest minimum-maximum range was in the Central region: 0.06 to 13.66 per 10,000 population. For field epidemiological investigators, the lowest minimum county density was also in the East region, with 0.01 per 10,000 population, while its maximum was 12.89. However, the widest minimum-maximum range was in the Central region: 0.06 to 13.66 per 10,000 population.

For all categories of PHWs, the absolute ratio between the highest and lowest county density in regions was larger than the ratio between the highest mean regional density (East) and the lowest (West). Overall inequality in the distribution of PHWs was mostly due to within-region inequality, accounting for 98.5% or more of overall inter-county inequality in the distribution of all categories of PHWs.

#### Province-level contribution to overall inequality

Table 3 shows the decomposition of inter-county inequality by province, which is different from that by region. Comparing between-region inequality, the absolute value of between-province inequality was more significant for the PHW categories. Between-province inequalities were all above 20%, while within-province inequalities were no less than 74%. For staff, the percentage contribution of between-province to overall inequality was 25.6% (0.0168 of 0.0657), whereas the contribution of between-region to overall inequality was 1.1% (0.0008 of 0.0657). The corresponding numbers for health professionals were 22.8% (0.0160 of 0.0702) compared with 1.5% (0.0010 of 0.0702, between-region contributions). For field epidemiological investigators, the corresponding numbers were 20.4% (0.0222 of 0.1088), compared with 0.5% (0.0005 of 0.1088, between-region contributions).

**Table 3:** Decomposition of inter-county inequality (*n* = 2712) by province for three categories of PHWs in China, 2012^a^

Workforce category	Inequality measure	Overall inter-county inequality	Within-province inequality	Between-province inequality	Within-province inequality (% of overall)^b^	Between-province inequality (% of overall)^b^
Staff	Theil T	0.0655	0.0489	0.0168	74.4%	25.6%
	Gini	0.2948				
Health professionals	Theil T	0.0702	0.0542	0.0160	77.2%	22.8%
	Gini	0.3038				
Field epidemiological investigators	Theil T	0.1088	0.0866	0.0222	79.6%	20.4%
	Gini	0.3788				

Abbreviation: PHW, public health workforce

^a^ Data source: National cross-sectional survey of the CDC system for 2012, Division of Disease Control and Prevention, Ministry of Health. County number is 2712 because missing data

^b^ Within-region inequality (% of overall) is the ratio of “within-region inequality” to “overall inter-county inequality”, and between-region inequality (% of overall) is the ratio of “between-region inequality” to “overall inter-county inequality”

**Fig. 1:**
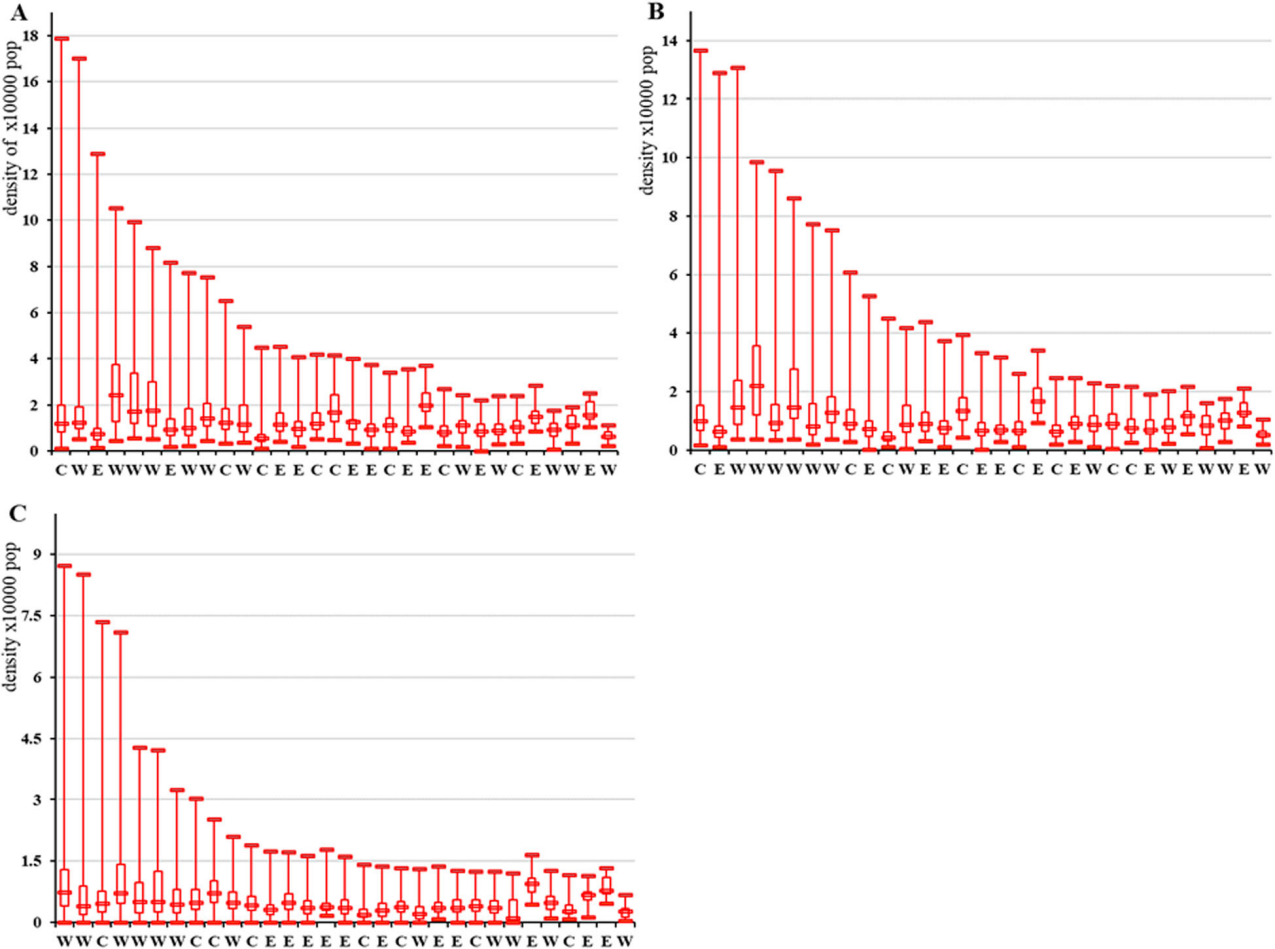
Box plots of the density of three types of workforce per 10,000 population by county share in province. Note: X axis = 31 provinces. Y axis = density of workforce per 10,000 population. Panel **A** = description for staff. Panel **B** = description for health professionals. Panel **C** = description for field epidemiological investigators. Because of the confidentiality of the data, we have hidden the names of the provinces and used the region code to represent them: E for the eastern provinces, C for the central provinces, and W for the western provinces. According to the range between maximum county density and minimum county density in each province, the provinces are sorted from large to small

Box plots in Fig. 1 show a wide variation of density in the three categories of PHWs per 10,000 population by county share per province. For staff, a central province had the absolute widest minimum-maximum range: 0.12 to 17.87 per 10,000 population. For health professionals, the absolute minimum-maximum range was also widest in the same province: 0.16 to 13.67 per 10,000 population. For field epidemiological investigators, the absolute minimum-maximum range was widest in a western province: 0.00 to 8.72 per 10,000 population.

Figure 1 provides each province’s mean density (median) of PHWs per 10,000 population. The highest mean provincial staff density was 1.74, and the lowest was 0.49. The highest mean provincial health professionals’ density was 1.48, and the lowest was 0.39. For field epidemiological investigators, the highest mean provincial health professionals’ density was 0.83, and the lowest was 0.29. The ratio between the highest and lowest county density in each province was larger than the ratio between the highest and lowest mean provincial density, so the within-province inequality was substantially larger than between-province inequality (Table 3).

#### Municipal-level contribution to overall inequality

Table 4 shows the decomposition of inter-county inequality by municipality, which differs from that by region and province. A comparison shows that between-municipality inequality was more than twice as large as between-province inequality for most categories of PHWs. The absolute value of between-municipality inequality for field epidemiological investigators is approximately 2.4 times as large as that between-province inequality.

**Table 4:** Decomposition of inter-county inequality (*n* = 2712) by municipality for three categories of PHWs in China, 2012^a^

Workforce category	Inequality measure	Overall inter-county inequality	Within- municipal inequality	Between- municipal inequality	Within- municipal inequality (% of overall)^b^	Between- municipal inequality (% of overall)^b^
Staff	Theil T	0.0655	0.0290	0.0365	44.3%	55.7%
	Gini	0.2948				
Health professionals	Theil T	0.0702	0.0323	0.0379	46.0%	54.0%
	Gini	0.3038				
Field epidemiological investigators	Theil T	0.1088	0.0627	0.0461	57.6%	42.4%
	Gini	0.3788				

Abbreviation: PHW, public health workforce

^a^ Data source: National cross-sectional survey of the CDC system for 2012, Division of Disease Control and Prevention, Ministry of Health. County number is 2712 because missing data

^b^ Within-region inequality (% of overall) is the ratio of “within-region inequality” to “overall inter-county inequality”, and between-region inequality (% of overall) is the ratio of “between-region inequality” to “overall inter-county inequality”

For staff, the percentage contribution of between-municipality to overall inequality was 55.7% (0.0365 of 0.0657), whereas the contribution of between-province to overall inequality was 25.6% (0.0168 of 0.0657). The corresponding numbers for health professionals were 54.0% (0.0379 of 0.0702) compared with 22.8% (0.0160 of 0.0702, between-province contributions). For field epidemiological investigators, the corresponding number was 42.4% (0.0461 of 0.1088, between-municipality contributions), which is the first result below 50%.

**Fig. 2:**
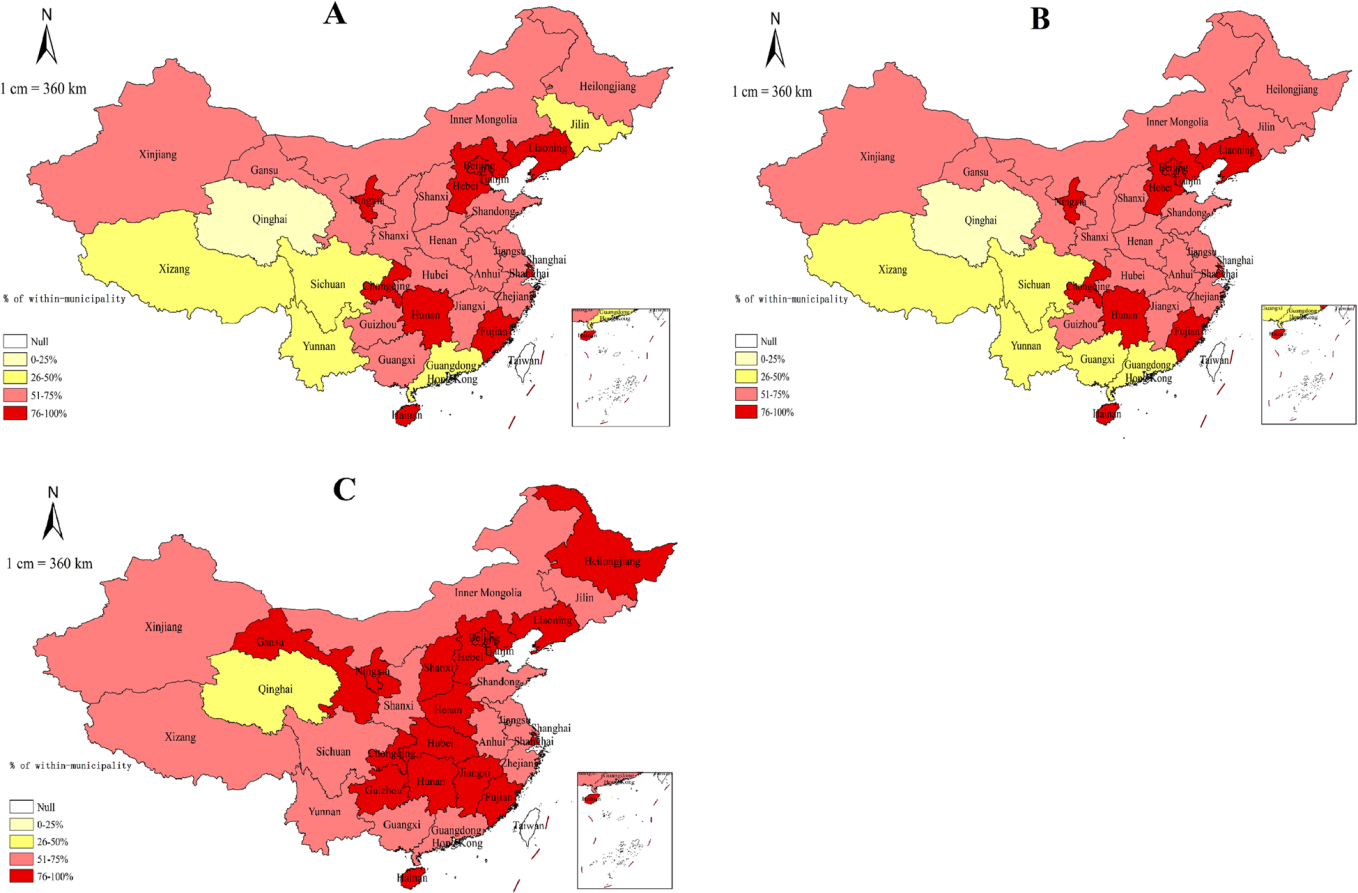
Distribution of the contribution of “within-municipality” inequalities in all 31 provinces by inner-county share. Different colors represent different contributions. Note: Panel **A** = contribution for staff. Panel **B** = contribution for health professionals. Panel **C** = contribution for field epidemiological investigators

We computed the decomposition of inter-county inequality by municipality in 31 provinces and used Chinese maps to show the contribution of within-municipality inequality (Fig. 2). However, the share of within-inequalities was different from the overall inter-county inequality. For staff, the absolute value of within-inequality in only six provinces was less than or equal to 50% among 31 provinces, while in other provinces, it was more than 50%. The situation of health professionals was similar. However, the situation of field epidemiological investigators was more serious. The within-municipality inequalities in almost all provinces contributed to more than 50%, with the exception of only one province.

### Contextual factors influencing inequality in PHWs

We also analyzed the contextual factors that affect within-municipality and between-municipality inequalities of PHWs. The analysis was conducted only at the municipal level, as the number (333) of municipalities was sufficient for a stable statistical analysis. As shown in Table 5, the results of *F*-test show that all linear regression equations have good significance. The linear regression model determined one factor that significantly correlated with within-municipality and between-municipality inequalities for all three categories of PHWs. The larger the agency building area per employee, the higher the proportion of PHWs. Per capita GDP had a similar effect, except for between-municipality inequality of professionals and within-municipality inequality of field epidemiological investigators. The agency government financial allocation per employee only influenced between-municipality inequality of staff. Numbers of kindergartens and primary schools per 10,000 population and numbers of training days per employee per year did not show a significant effect.

**Table 5:** Contextual factors associated with ‘between’ and ‘within’ municipal inequality for three categories of PHWs in China, 2012

	Staffs				Health professionals				Field epidemiological investigators			
	Between		Within		Between		Within		Between		Within	
contextual factors	Parameter estimates B(95%CI)	*P*^b^	Parameter estimates B(95%CI)	*P*^b^	Parameter estimates B(95%CI)	*P*^b^	Parameter estimates B(95%CI)	*P*^b^	Parameter estimates B(95%CI)	*P*^b^	Parameter estimates B(95%CI)	*P*^b^
Per capita GDP	0.0000 (0.000–0.000)	0.000	0.0000 (0.000–0.000)	0.001	0.0000 (0.000–0.000)	0.606	0.0000 (0.000–0.000)	0.012	0.0000 (0.000–0.000)	0.000	0.0000 (0.000–0.000)	0.122
Numbers of kindergartens and primary schools per 10,000 capita	0.0002 (0.000–0.001)	0.314	0.0005 (0.000–0.001)	0.140	0.0002 (0.000–0.001)	0.524	0.0004 (0.000–0.001)	0.182	0.0000 (0.000–0.000)	0.978	0.0000(−0.001–0.001)	0.986
Agency government financial allocation per employee	−0.0023(− 0.004--0.001)	0.012	− 0.0005(− 0.004–0.003)	0.748	−0.0024(− 0.005–0.001)	0.105	−0.0007(− 0.003–0.002)	0.622	0.0002(− 0.002–0.002)	0.811	− 0.0010(− 0.005–0.003)	0.570
Agency building area per employee	− 0.0015(− 0.002--0.001)	0.000	−0.0014(− 0.002--0.001)	0.001	0.0014 (0.001–0.002)	0.000	− 0.0011(− 0.002–0.000)	0.001	−0.0013(− 0.002--0.001)	0.000	−0.0016(− 0.002--0.001)	0.000
Numbers of training days per employee per year	0.0006(−0.002–0.003)	0.636	0.0035(− 0.001–0.008)	0.144	−0.0021(− 0.006–0.002)	0.348	0.0035 (0.000–0.007)	0.085	0.0016(− 0.001–0.005)	0.303	0.0000(− 0.005–0.005)	0.990
R-square	0.247		0.112		0.099		0.088		0.163		0.061	
F-value	17.85		6.869		5.958		5.226		10.593		3.519	
*P*	0.000		0.000		0.000		0.000		0.000		0.002	

^a^ Data source: National cross-sectional survey of the CDC system for 2012, Division of Disease Control and Prevention, Ministry of Health

^b^ All the regression estimates are adjusted for the effect of region (East, Central, West)

## Discussion

This is the first study to use the smallest geographical units to measure the inequality of PHWs in China’s CDCs. Through the gradual decomposition of regions, provinces, and municipalities by inner-county share, this study revealed the extent, nature, and sources of misdistribution of the PHW throughout China. This is also the first study on the distribution of the PHW 10 years after China’s SARS outbreak. This study is significant in that it contributes to helping Chinese governments at all levels and health administrations to develop PHW allocation policies related to geographical and structural equity.

In this study, the Gini index was used to reflect the overall inequality, while the Theil index was mainly used to decompose the sources of inequality. The overall inequality results show that the inequality of health professionals and field epidemiological investigators was worse than that of staff, with the two being a subset of staff. Among them, field epidemiological investigators had the worst inequality, with a Gini coefficient of close to 0.4 (warning limit for high inequality), which is consistent with the real-life conditions in China [25]. According to a qualitative survey conducted by the Chinese Center for Disease Control and Prevention, field epidemiology training programs (FETP) started in China in 2001, and although such programs have been successful in many areas, the effectiveness and quality of these training programs vary by region. The primary reasons for the lack of success in this regard are limited financial support; inadequate attention from those in leadership roles; absence of good instructors; and only partial involvement of students in field practice training due to excessive routine work [26]. Therefore, local governments in underdeveloped areas should increase financial input, emphasize and support the development of training projects, and enhance trainers’ enthusiasm and motivation through various incentives, thereby ultimately increasing the number of field epidemiological investigators in their jurisdictions.

Furthermore, research has shown that the decomposition of overall inequality into within-group and between-group inequality is important for identifying the sources of inequality [19, 27]. Contribution rate can improve the understanding of the causes of inequality in the allocation of PHWs. From the perspective of inequality decomposition, we found that PHWs in China were unevenly distributed across geographical areas, including regions, provinces, and municipalities. The contribution of within-region inequality has an overwhelming superiority, although there are differences between regions in terms of comparative analysis of data concerning basic characteristics. This suggests that the imbalance of PHWs is internal to each region, including all three categories of PHWs. Further, the contribution of inequality within-province also has significant weight, suggesting that although provinces are smaller geographical partitions than regions, the PHW inequities are greater within each province. This supports the results of the decomposition by describing the density of PHW. For the density of provinces, the difference between the averages of PHWs was smaller than the difference between the maximum density and the minimum density of counties within each province, and the difference within provinces was greater than that between provinces. The subsequent decomposition of inter-county inequality by municipality also supports this point. The overall decomposition of inter-county inequality (by municipality) shows that staff and health professionals have a similar proportion of within-municipal and between-municipal inequality. However, the results of the decomposition of inter-county inequality by municipality of 31 provinces indicates that within-municipality inequalities still cause the municipal inequality of most provinces. This suggests that the government, especially at the municipal level, should focus on eliminating PHW inequalities between counties within their jurisdictions. The finance and human resources departments of the government should introduce various incentive policies, such as signing contracts with medical colleges to train medical students to serve at the basic level or improving the salary level of basic public health workers, in order to attract more talents to county-level CDCs with weak human resource allocation.

The regression analysis revealed the contextual factors affecting the geographical distribution of PHWs, which included living environment, working conditions, personal development, and children’s education. For the first time, large-scale and representative data sets were used to measure the role of these factors in explaining the inequality of PHW distribution in China. Agency building area per employee caused between-municipality and within-municipality inequalities for all three categories of PHWs. Per capita GDP had a similar effect, except for between-municipality inequality of professionals and within-municipality inequality of field epidemiological investigators. Therefore, our results reveal that the key incentives for the unequal distribution of PHWs among municipalities are the level of infrastructure construction of institutions and the level of economic development of residential areas. A good level of infrastructure construction means that PHWs can have a better and more spacious working environment, which may be an aspect that PHWs pay more attention to. The level of economic development in the residential area is equally important, because it means that the local development can provide a better living environment and facilities for themselves and their family.

The results demonstrated that inequality differences in China’s PHW allocation between regions and between provinces are small, but inequality differences between counties at the provincial or municipal level are larger. This suggests that a regional inequality elimination strategy is not applicable and that China needs a PHW configuration strategy focusing on provinces and municipalities. This is consistent with China’s current economic and social development. As the Chinese government recently indicated, imbalances in intra-regional development have become a social contradiction in China [12]. For example, most of China’s provinces have national and provincial poverty-stricken counties, even in some developed provinces of eastern regions. According to the latest national poverty-stricken counties list, Guangxi and Hebei province, which belong to the eastern regions, have 22 and 13 national poverty-stricken counties, respectively [28]. Whether it is economic development or resource allocation, within-province differences are greater than between-province differences. At present, China attaches great importance to poverty alleviation. In terms of CDC resource allocation, poverty alleviation policies also need to be formulated, which is part of the national health poverty alleviation strategy. The government must take financial, material, and human resource measures to strengthen the support for CDCs at disadvantaged county-level units and enhance their ability to attract talents.

In addition, we also found that our inequality results were worse than those of research using traditional geographical units [29, 30], using both Gini and Theil T. These results suggest that the smaller the geographical units, the higher the degree of inequality (other factors remaining unchanged) [19]. Further, the consistency between density analysis and inequality decomposition verified that inequality decomposition is effective. Inequality decomposition is a simple and effective method to analyze the source of inequality and can be used in similar research.

This study has several limitations. First, although the study used data quality control and investigator training, insufficient data collection was inevitable due to objective reasons. However, when we compared the data to the health statistics yearbook, the differences were negligible. Second, the equity analysis of the distribution can only tell us the quantity distribution of PHWs but cannot reflect the differences in personnel quality. However, in the descriptive analysis part, we were able to show some problems in the quality distribution of PHWs by analyzing the regional distribution differences of the three types of PHWs. As the core professionals, the distribution of field epidemiological investigators is the most inequality, suggesting that quality distribution of PHWs may have a potential greater inequality. Third, although our data are derived from national surveys and are broader than those available from the public sector, due to the conservative nature of data disclosure in the Chinese administration, our data include a reporting gap. The availability of data on health workers is one of the current major obstacles to conducting health workforce research and developing appropriate health worker policies [31]. As far as we know, the data and information of all county CDCs in China are insufficiently disclosed to the public sector. Data availability has a direct impact on PHWs’ distribution decisions, especially at the local level.

## Conclusions

This study looked for sources of inequality between PHWs through decomposition of different geographical units among regions, provinces, and prefecture-level cities. The successive decomposition showed that inequality is mainly concentrated in counties at the within-province and within-municipal levels. Addressing the inequality of health professionals, especially field epidemiological investigators who play a crucial role, must be prioritized, as this relates to the realization of the basic functions of the CDC. Moreover, the results clearly suggest that the government, especially municipal governments at the provincial level, should increase financial investment in CDCs with worse resource allocation in their jurisdiction through various compensation and incentive mechanisms, enhance their infrastructure, and improve the salary of personnel in these institutions, in order to attract more public health professionals to these institutions, thereby fundamentally resolving the problem of unequal distribution of PHWs.

## Data Availability

This survey was administered through a collaboration with the National Health Commission of the People’s Republic of China (the former Ministry of Health [MOH]), and the data ownership belongs to the former MOH. We received the requested data for analysis from this agency. Interested parties could contact the National Health Commission to request access to data.
